# Editorial: Assessing the Therapeutic Uses and Effectiveness of Virtual Reality, Augmented Reality and Video Games for Emotion Regulation and Stress Management

**DOI:** 10.3389/fpsyg.2019.02763

**Published:** 2019-12-11

**Authors:** Federica Pallavicini, Stéphane Bouchard

**Affiliations:** ^1^Department of Human Sciences for Education “Riccardo Massa”, University of Milano Bicocca, Milan, Italy; ^2^Département de Psychoéducation et de Psychologie, Université du Québec en Outaouais, Gatineau, QC, Canada

**Keywords:** virtual reality, emotion, emotion regulation, video game, stress, stress management

Virtual reality (VR), Augmented Reality (AR), and Video games (VGs), because of their reasonable cost and increasing diffusion among the public, are becoming very interesting and promising therapeutic approaches for improving individuals' health and well-being (e.g., Granic et al., 2014; Giglioli et al., 2015; Riva et al., 2016; Hemenover and Bowman, 2018).

However, despite that numerous scientific studies have demonstrated the therapeutic benefits of these technologies for diverse cognitive functions and individual patients (e.g., Parsons et al., 2017; Bediou et al., 2018; Bouchard and Rizzo, 2019; Riva et al., 2019), less attention has been devoted to the therapeutic use of such tools for the assessment and training of emotion regulation and stress management skills.

Therefore, we brought together within this Research Topic contributions from researchers investigating theoretical, empirical, experimental, and case studies of VR, AR, and VGs for emotion regulation and stress management assessment and training. This Editorial will provide an overview of the articles accepted for publication in the Research Topic.

## VR and VGs Content and Application for Emotion Regulation and Stress Management

Pizzoli et al. addresses the important question of how to build effective VR contents to promote relaxation and decrease stress. The authors explores a new theoretical approach, that would be based on VR with personalized content, grounded on user research to identify important life events and on the rendering of such events with symbols, activities, or other virtual environments contents. According to the authors, it is possible that such an approach would obtain more sophisticated and long-lasting relaxation in users.

Lindner et al. explore the potential of consumer-targeted VR relaxation applications for widespread dissemination. In their study, they analyze “real-world” aggregated uptake, usage, and application performance statistics from a first-generation consumer-targeted VR relaxation application (i.e., the Happy Place) which has been publicly available for almost 2 years. According to their findings, primarily user engagement needs to be addressed in the early stage of development by including features that promote prolonged and recurrent use (e.g., gamification elements).

Pallavicini et al. describe results of a systematic review on the impact of video games training on emotional regulation and stress management skills—as well as on cognitive abilities (i.e., processing and reaction times, memory, task-switching/multitasking, and mental spatial rotation)—in the healthy adult population. According to the results, non-commercial video games as well as commercial video games can be effective in inducing positive emotions and in reducing individual levels of stress in healthy adults. Furthermore, results showed that the number of studies conducted about video games training on emotional regulation and stress management skills (i.e., 5) is still smaller than the amount of studies related to cognitive training (i.e., 30).

## Applications of VR for Emotion Regulation and Stress Management Assessment and Training in Mental Health Contexts

Wechsler et al. report results of a systematic review focused on the comparison of VR and *in vivo* exposure in studies applying an equivalent amount of exposure for phobic anxiety disorders and their treatment. According to the 9 studies included, VR exposure show a higher potential and is not less effective than *in vivo* exposure in specific phobia and agoraphobia.

Tarrant et al. carried out a pilot study aimed to examine changes in brain patterns associated with the use of VR for anxiety management in people with generalized anxiety disorder (GAD). The study involved 14 patients suffering from GAD. Results showed that both a quiet rest control condition and the VR meditation significantly reduced subjective reports of anxiety. However, the VR intervention uniquely resulted in physiological reduction of anxiety.

Atzori et al. and Atzori et al. describe results of a randomized controlled trial that tested the effectiveness of VR as a distraction technique to help control pain in children and adolescents undergoing venipuncture. Fifteen patients suffering from oncological or hematological diseases were randomly assigned to the VR (i.e., SnowBall) or the no-VR control condition (i.e., standard of care). Results showed that VR was more effective in distracting patients during venipuncture and it elicited more positive emotions than the traditional distraction technique.

The same authors report results of a pilot study aimed to evaluate the feasibility and effectiveness of immersive VR as an attention distraction analgesia technique for pain management in children and adolescents undergoing painful dental procedures. Five patients received tethered immersive interactive VR distraction during one dental procedure. On a different visit to the same dentist (e.g., 1 week later), each patient also received a comparable dental procedure during the control condition “treatment as usual.” Findings showed that patients reported significantly lower “worst pain” and “pain unpleasantness,” and had significantly more fun in the VR condition, compared to a comparable dental procedure without VR.

Kip et al. describe results of a study aimed to explore why and in what way VR can be of added value for treatment of forensic psychiatric patients. Based on the results of semi-structured interviews conducted with 8 therapists and 4 patients, 6 scenarios about possibilities for using VR in treatment were created and presented to 89 therapists and 19 patients in an online questionnaire. According to the analysis of the qualitative data emerged, VR offers a broad range of possibilities for forensic mental health, including the training of emotion regulation skills (i.e., coping skills that support the patient in not giving in to impulses when confronted with difficult, emotion-eliciting situations, or stimuli).

Cebolla et al. report results of a randomized controlled trial aimed to investigate the efficacy of a self-compassion meditation procedure based on the machine to be another (TMTBA) system—which uses multi-sensory stimulation to induce a body swap illusion—in increasing positive affect states, mindful self-care, and adherence to cognitive behavioral interventions (CBIs). Sixteen participants were randomly assigned to two conditions: TMTBA-VR and usual meditation procedure (CAU). Results showed that after 2 weeks, both conditions showed a similar frequency of meditation practice and increases in specific types of self-care behaviors, with the frequency of clinical self-care behaviors being significantly higher in TMTBA. According to the authors, embodied VR could be an interesting tool to facilitate and increase the efficacy of CBIs by facilitating the construction of positive and powerful mental images.

## Theoretical Perspectives of Understanding VR in Emotion Regulation and Stress Management Assessment and Training Programs

Gromer et al. report results of a study in which they experimentally manipulated presence and fear to unravel the causal link between these responses in VR environments. A sample of 49 fearful participants were immersed into a virtual height situation and a neutral control situation (fear manipulation) with either high vs. low sensory realism (presence manipulation). Ratings of presence and verbal and physiological (skin conductance, heart rate) fear responses were recorded. Results showed that experiencing emotional responses in a virtual environment leads to a stronger feeling of being there, i.e., increase presence. In contrast, the effects of presence on fear seem to be more complex: on the one hand, increased presence due to the quality of the virtual environment did not influence fear; on the other hand, presence variability that likely stemmed from differences in user characteristics did predict later fear responses.

## Author Contributions

All authors listed have made a substantial, direct and intellectual contribution to the work, and approved it for publication.

### Conflict of Interest

SB is a consultant to and own equity in Cliniques et Développement In Virtuo, a spin-off from the university that uses virtual reality as part of its clinical services and distributes virtual environments. The terms of these arrangements have been reviewed and approved by the Université du Québec en Outaouais in accordance with its conflict of interest policies. The remaining author declares that the research was conducted in the absence of any commercial or financial relationships that could be construed as a potential conflict of interest.
